# “*Caballo*”: risk environments, drug sharing and the emergence of a hepatitis C virus epidemic among people who inject drugs in Puerto Rico

**DOI:** 10.1186/s12954-020-00421-z

**Published:** 2020-10-23

**Authors:** R. Abadie, K. Dombrowski

**Affiliations:** 1 Department of Anthropology, University of Nebraska-Lincoln, 839 Oldfather Hall, Lincoln, NE 68588 USA; 2 Department of Anthropology, University of Vermont, 72 University Place, Burlington, VE 05405 USA

**Keywords:** PWID, HCV, Drug sharing, Risk environment, Harm reduction, Puerto rico

## Abstract

**Background:**

Sharing drug injection equipment has been associated with the transmission of HCV among PWID through blood contained in the cooker and cotton used to prepare and divide up the drug solution. While epidemiologists often subsume this practice under the sharing of “ancillary equipment,” more attention should be paid to the fact that indirect sharing takes place within the process of joint drug acquisition and preparation.

**Methods:**

We employed an ethnographic approach observing active PWID (N = 33) in four rural towns in Puerto Rico in order to document drug sharing arrangements involved in “caballo”, as this practice is locally known. We explored partners’ motivation to engage in drug sharing, as well as its social organization, social roles and existing norms.

**Findings:**

Findings suggest that drug sharing, is one of the main drivers of the HCV epidemic in this population. Lack of financial resources, drug packaging, drug of choice and the desire to avoid the painful effects of heroin withdrawal motivates participants’ decision to partner with somebody else, sharing injection equipment—and risk—in the process. Roles are not fixed, changing not only according to caballo partners, but also, power dynamics.

**Conclusion:**

In order to curb the HCV epidemic, harm reduction policies should recognize the particular sociocultural contexts in which people inject drugs and make decisions about risk. Avoiding sharing of injection equipment within an arrangement between PWID to acquire and use drugs is more complex than assumed by harm reduction interventions. Moving beyond individual risk behaviors, a risk environment approach suggest that poverty, and a strict drug policy that encourage users to carry small amounts of illicit substances, and a lack of HCV treatment among other factors, contribute to HCV transmission.

## Background

While HCV-related deaths in the United States seem to have declined recently [1], research suggests the existence of an emerging hepatitis C virus (HCV) epidemic among people who inject drugs (PWID) in non-urban areas [2], which is likely associated with the transition from oral prescription opioid use to injection and often with the transition from prescription opioids to heroin [3]. Rates of HIV and HCV among PWID are on a divergent trajectory. HIV prevalence has been declining for almost a decade, while HCV has increased over the same period [4].

Nowhere in the United States are these trends more prevalent than in Puerto Rico, a US territory, where recent epidemiological data suggest epidemic levels of HCV among PWID, with 89% prevalence in metropolitan San Juan and 78.4% in rural areas [5, 6]. In the US, HCV prevalence among PWID ranks highest in Puerto Rico, at a similar level as the worst cases in the global south [7, 8].

Epidemiological studies of HCV transmission routes among PWID show that the virus can be transmitted not only though blood contained in shared syringes but also by sharing the cooker and cotton used to prepare the drug solution [9–11]. Yet, epidemiologists often misunderstand the dynamics of HCV transmission among PWID, subsuming the common use of a cooker, or a filtering cotton ball, as the shared use of “ancillary equipment” [12, 13]. As social scientists have noted, equipment sharing is often a proxy for preparing and dividing drugs [14, 15]. Based on a study of PWID in Colorado, Koester (2005) suggests that indirect sharing, which happens when powder drugs are diluted in water before been divided up in a cooker with the help of a syringe, is an efficient way of distributing drugs among injection partners. Using the calibration on the syringe barrel allows participants to compare the syringe contents’, effectively ensuring an equitable distribution. This type of indirect sharing which happens more often than direct syringe sharing, when one syringe is shared from one user to another [16] (Friedman et al. 1997) has been found to be a common feature among PWID in a variety of social contexts [17–24]. In a social network study of PWID in Brooklyn, New York Curtis and colleagues (1995) found that indirect sharing is extensively practiced within a network particular social networks, contributing to define a user position in it. Those that engage in drug sharing often, are the “regulars” or insiders who are placed at the center of the network, while those that do engage occasionally sometimes coming from other neighborhoods or sporadically over the weekends, tend to occupy a peripheric position [25]. A similar study by Zule with PWID in San Antonio shows that participants drug sharing arrangements are asymetric relationships, with the person that provides the drug in a position to direct drug preparation and the person receiving the drug solution unable to avoid the indirect sharing of injection equipment [26].

A qualitative study of PWID in Tajikistan shows that while few users report direct syringe sharing, joint drug acquisition and preparation is common, particularly in outdoor settings or if participants experience withdrawal symptoms [27]. Another study in Viet Nam shows that drug sharing practices are driven by heroin price and accessibility as well as a punitive approach to drug use that penalizes drug possession [28].

In a multi-year study of Puerto Rican PWID in New York City and Bayamon, in San Juan found that, recent migrants from the island exhibit a much higher frequency of indirect drug sharing than native users [29]. An ethnographic study conducted among PWID in San Juan extends these observations by exploring the motivations and social roles involved in drug sharing arrangements [30]. Finlinson (2011) shows that the price and packaging of drugs and access to drug preparation materials along with power differences among partners shape the process by which drugs are prepared and injected. Other ethnographic studies demonstrated that drug sharing is facilitated by the type of heroin available. For example, Bourgois documents how homeless PWID in San Francisco use “black tar,” a sticky variety of heroin, originating in Mexico, that is extremely hard to divide up without preparing it, which, in turn, encourages the sharing of injection equipment [31, 32].

Departing from epidemiological views that focus on individual behaviors, these authors make a valuable contribution to the understanding of HCV risk among PWID by showing how particular social contexts, from the ways in which drugs are acquired, to social roles, cultural norms, drug policies and power dynamics among those involved in “*caballo”* shape HCV risk. These findings complicate traditional epidemiological views shaping harm reduction initiatives, suggesting that forms of indirect sharing within the process of jointly acquiring and using drugs, are not easily modified by knowledge about HCV transmission, or the access to new injection equipment alone. As critics have suggested, the focus on individual behaviors at the expense of local social contexts in which PWID live and make decisions about risk, might obscure how social, structural and environmental contexts shape drug use and related harms [33–37].

Nested within a study of social networks and HIV/HCV risk among PWID in rural Puerto Rico, we propose an ethnographically informed approach to “*caballo”,* the joint acquisition and sharing of drugs, as a window into the social production of an HCV epidemic among PWID. While drug sharing arrangements among PWID have been amply documented, an ethnographic study of caballo in Puerto Rico will illuminate the social context behind the joint acquisition and use of drugs and its related epidemiological risk.

## Methods

This paper utilizes ethnographic data from PWID recruited into a multi-phase study of social networks and HIV/HCV risk in four rural towns in the mountainous area of central Puerto Rico. In the first phase of this study we collected demographic and sociometric data on PWID in rural Puerto Rico, as well as information on injection behaviors, particularly on the sharing of syringes and injection equipment. In addition, rapid blood testing for HIV and HCV were conducted using INSTI Rapid HIV antibody tests (Biolytical Laboratories) and OraQuick HCV Rapid antibody tests (OraSure Technologies).

It was during the administration of a National Health Behavioral Survey (NHBS) questionnaire [38] that we learned for the first time about “*caballo”*. Consistently, we heard from participants that while they carried their own syringe and avoided sharing it with others, they could not avoid using the same cooker within the process of drug sharing. Participants described the ways in which they divided up the drug solution in the cooker, using a backloading method to distribute it among partners. All participants insisted that they “always used their own cooker” during the preparation. Of course, since there are two or more caballo partners and only one cooker available, this was not possible. After this phase of data collection, we were left with many questions about the social organization and the meaning of drug sharing among this population.

To explore these issues, we conducted an ethnographic study with (N = 33) participants randomly recruited from the first phase. During the participant observation, each participant was followed -with their consent- for up to two weeks, documenting practices related to joint drug acquisition and use. Yet, our ethnographic observations were broadly constructed, focusing not only on drug sharing practices, but in describing their everyday lives and the strategies they used in order to afford their drugs of choice. For example, we had ample opportunity to observe participants’ hustling, in car washes, or guarding a parking lot, or begging at the entrance of banks or dollar stores or other venues with heavy foot traffic. After partnering with *El* *Punto* *en* *la* *Montana,* the only Syringe Exchange Provider in the area, we earned participants’ trust and were allowed to join them at the “chutin” a Spanglish deformation of shooting gallery. In so doing, we were able to collect data on their drug sharing practices. We paid particular attention to the ways in which caballo partners talked about acquiring and sharing drugs in an attempt to convey the norms or hidden scripts regulating caballo arrangements. In addition, we conducted in depth interviews to understand how PWID in our study described the social practice of “caballo”, the joint acquisition and later use of drugs. Some of the questions we posed were what is caballo, when do participants do it and with whom. We used our initial observations to iteratively refine our research questions. Since we had observed during the course of the ethnographic data collection that sometimes arguments emerge during caballo, we asked participants about potential problems or conflicts associated with this practice and we also inquired about participants’ views on drug sharing related HCV risk and their perceived ability to enact changes to prevent its transmission.

One limitation of our data collection is that for security reasons, ethnographic observations were conducted during the day and until dusk and only during working days. Abandoned and dilapidated houses without electricity, shooting galleries are seldom used at night but are more used early in the day -as soon as drug selling spots are open- and on weekends when “regulars’ are joined by more occasional users. Despite the limits imposed by fieldwork dynamics we believe that our observations of caballo and the social context in which it occurs were not affected by these constraints.

Fieldnotes were transcribed while in-depth interviews were transcribed and translated. All personal identifiers were removed. MAXQDA software was employed to manage coding. Codes were developed to convey the wide arrange of themes in the data set: caballo, drug acquisition, motivations, social roles, norms and expectations and injection setting, among others. These codes were then iteratively revised and re-organized until they represented higher-level axial codes describing participants’ caballo experiences [39, 40]. Following Strauss’ grounded theory approach [41] (Strauss and Corbin 1998) the interpretation of the data emerged intuitively without imposing a pre-existing theoretical framework.

The study received IRB approval through the (omitted due to blind review) and the (omitted due to blind review). Participants provided written consent at the study office prior to enrollment in the study and were compensated for their time and travel expenses.

## Findings

Participants’ sociodemographic background (Table 1).

**Table 1:** Descriptive statistics

	Mean/%	SD	N
Age (years)	44.15	9.12	33
% Male	87.88%		33
% Heterosexual	96.97%		33
% Unemployed	75.75%		33
% Experienced homelessness in past year	21.21%		33
% Graduate high school (or higher)	54.55%		33
% Married or living together as married	21.24%		33
% Annual income < $5000	51.52%		33
Average money spent on drugs daily	$41	$24.45	33
% Currently covered by health insurance	90.91%		33
% Ever participated in a drug treatment program	15.15%		33
Number of years injecting	20.91	10.54	33
Age at first injection	22.27	7.97	33
% Injected 4 or more times per day	30.30%		33
% Injected 2–3 times per day	39.39%		33
% Tested positive for HIV	3.03%		33
% Tested positive for HCV	84.84%		33
Last time you injected with someone did you…			
Use a needle after someone else	7.14%		28
Use a sterile needle	84.62%		26
Use a cooker, water, or cotton that someone else had used	71.43%		28
Use drugs that had been divided with a syringe that someone else had used	32.14%		28

Participants had a mean age of 44.15 years and the sample had a standard deviation of 9.2 years. The sample is overwhelmingly male (87.8%) and heterosexual (96.97%). Three-quarters were unemployed at the time, and almost one-half lived in poverty and had completed high school or a higher educational level. One in five were married or living together and the same number had been homeless during the past year. Almost all participants (90.91%) were currently covered by health insurance, with a large majority having “La Reforma,” the local version of Medicare/Medicaid. Around one in six (15.15%) had participated in a drug treatment program but only one participant had enrolled during the past year. Participants had been injecting for a mean of 22.91 years with a standard deviation of 10.54. Age at first injection is 22.27 years with a standard deviation of 7.97. Almost four out of ten reported injecting two or three times a day while one in three injected four times or more a day. While HIV prevalence in this population is extremely low (3.03%), HCV prevalence reaches epidemic levels, with almost nine out of ten study participants testing positive reactive for the virus (84.8%). Less than one in ten (7.14%) declared having used a syringe after somebody else had used it the last time they injected with somebody, and a large majority (84.6%) admitted using a sterile syringe. Almost three-quarters (71.43%) used a cooker, cotton, or water that somebody had previously used, while one in three (32.14%) divided drugs with a syringe that had been previously used by somebody else.

## *Caballo* and the social production of an HCV epidemic

In rural Puerto Rico, two or more PWID often pool funds necessary to acquire and later share drugs. Most participants in our study have as a drug of choice heroin “droga” and cocaine “perico”, “speedball”. Speedballs have more heroin than cocaine, a usual way in which participants talk about their drug mix is by identifying it by the ratio of heroin to cocaine. For example, they would say “1–2” meaning one bag of cocaine and two of heroin. Other users might prefer three bags of heroin and one of cocaine “1–3”. In turn, this preference is also reflected in drug sharing arrangements. The drugs are mixed together in a cooker dissolved in water, and the resulting drug solution is shared usually through backloading, removing the plunger in a syringe and squirting the content using the tip of the needle of a loaded syringe, before placing it back. This practice is locally known as “*caballo*” (literally, horse). Participants do not recall the origin of the name, *“caballo”* but suggest that the same expression is used on the island in situations where people pool resources to acquire and later consume goods together, usually food but also transportation. An ethnographic fieldnote taken at the shooting gallery a few blocks away from where our office was located, describes two study participants, Pablito and Cesar Cayey engaging in a *“caballo”:*

Pablito and Cesar had bought their drugs of choice at the one of the local *“Puntos”*, the drug selling spot, only a few blocks away from the dilapidated house that served as shooting gallery. They briefly discussed how the drugs would be divided up agreeing that since they had contributed the same amount of money, each would receive equal parts of the drug solution. However, since Pablito, was helping Cesar inject because his partner was unable to find his own veins, Pablito would be in charge of the preparation.

The cooker, a small sized tin cup provided by the local Syringe Exchange Provider, *El* *Punto* *en* *la* *Montana* and a plastic water bottle were laid out on the cement floor by Pablito, who was in charge of the preparation. The plastic clear blue bag containing cocaine was opened and placed carefully on the cooker along with the contents of three colored metallic paper envelopes with heroin. Water from a plastic bottle was loaded into a syringe and then discharged into the cooker. The content was then mixed with the back of a syringe making sure it was completely diluted. Although the cocaine is a white powder and the heroin a light brown, grey or creamy color, when mixed with water, the solution turned into a brown color.

Without heating—it is believed to “eat up” the drug because when heated some of the content evaporates leaving a more powerful concentration, but less quantity-Pablito skillfully “recogio” or loaded the preparation with the help of a syringe with its needle inserted into a small cotton ball no larger than the head of a match to filter any impurities. With the syringe “full”, half of its content was then “echado” distributed into Cesar’s syringe by removing the plunger on the back. After verifying that both syringes had exactly the same amount by holding them side by side, using the lines on the side of the syringes as a guide, the plunger in Cesar’s syringe was placed back. Both syringes were now, half full, having divided a loaded syringe used to divide drugs up, in two equal parts.

Far from a ritual practice to strengthen a bond between injection partners, *caballo* seems to be a consciously made strategic decision to maximize drug access among PWID. Drugs are jointly acquired and then divided up among pooling partners. The most common arrangement is to distribute the drug solution according to the monetary contributions of each member proportionally. Partners can share drugs at *“brazo* *partido”* a local expression translated as half and half, or 50%-50%, if they contributed equally, although it is possible that the partner that contributes the most agrees to divide drugs equally, particularly if they have a long-standing relationship with the caballo partner.

Josephine, a 34-year-old woman who started injecting in her teens, provides a description of the advantages of this practice: “Look, let’s suppose that I want to use two and one [two bags of heroin and one bag of cocaine] and that you have $5 and I have $10. So, I ask you, Julio, ‘Do you have $5?’ ‘Yes,’ [you respond].[I say,] ‘Great! Let’s do two and one, you put in those $5 for the *perico* [cocaine] and I put [in for] the heroin.’ We put everything together in the cooker, and then we divide it in the syringe, half and half, and we get cured. That’s it.”

While most PWID in the study would prefer to avoid *caballo* if they could, particularly, for high frequency users, the economic demands make it extremely hard to go during the day without partnering with another user to acquire and use drugs. With a large proportion in unemployment, receiving meager social security checks, working at the lower levels of the local drug trade, or engaging in side hustles, it becomes extremely difficult for PWID in our area to secure the whole amount every time they need to inject. Opioid users dread the painful effects of heroin withdrawal, or what they call “being sick,” characterized by bodily pain and discomfort, nausea, coldness, shivers, and diarrhea that leave them “unable to function.” Only “la cura”, the cure, another dose of heroin, would stop or prevent these symptoms form occurring. Faced with limited resources to “get cured” the user has to make a choice between partnering with somebody in a *caballo* or going it alone and hustling until they can afford the whole dose they need. Entering into a caballo arrangement, enables them to feel normal again, while they can keep hustling to get their next dose. While the rewards of going alone might be higher because participants get their full dose, so are the associated costs because users have to battle their withdrawal symptoms while they come up with the money. And the longer it takes for participants to secure the resources to afford their dose, the worst their withdrawal symptoms become, offering a powerful incentive to enter into a drug sharing agreement with another user.

To avoid heroin withdrawal, if possible, caballo partners prefer to acquire their drug of choice in a Punto close to where they can use it, without delay. But in relatively small rural locations, drug choices are restricted, particularly in relation to package size. Locally, heroin can be bought in bags of five or ten dollars and cocaine comes in “*cinquillos*” fives, or $5, but if caballo partners can get a hold on a car, they usually pool up $2 or $3 each for gas and head to nearby Caguas o San Juan where bags cost the same but are two or up to three times larger. This drug packaging makes the trip worthwhile, even considering the gasoline costs and that time spent on the hour round trip could have been used “*revuleando*”, hustling, at home.

The frequency of *caballo* changes from participant to participant; some engage in *caballo* almost every time they use, while others do it less frequently. Since caballo partners tend to inject smaller drug amounts, they also need to engage in this practice more often. Speedball also provides more opportunities to share drugs because one partner might have cocaine but not heroin, while other might be in the opposite situation, in this situation, one solution is pooling resources and then dividing up the drug solution.

PWID in our study declared that they prefer not to do *caballo* for their first dose of the day, as it would significantly decrease the amount of heroin received and thus stave off withdrawal symptoms for a shorter amount of time. Walter, a 48-year-old user who injects two or three times a day, explains this choice with the use of a metaphor: “It’s like pizza: if you eat two pieces you are going to feel full but if you eat only one, or just a bite, you will feel hungrier sooner.” However, here economic considerations play a role. If access to a sufficient dose to prevent the onset of withdrawal symptoms is not available, users might resort to *caballo* early in the day. Those who have been successful in securing their own first dose might decide to engage in *caballo* later in the day in order to maximize resources, and can afford to engage in the practice more selectively. Caballo is also affected by a particularly punitive version of the war on drugs adopted by the Island since the 1980′s. Most of our study participants have been jailed at least once in their lifetime, often for non-violent drug offences [42]. Having no more than a few bags can lead to heavy prison sentences under the “possession with intent” to distribute charges. To avoid problems, PWID tend to carry small drug amounts with them, which, in turn, encourages drug sharing.

*Caballo* can be structured along defined social roles, with important epidemiological repercussions. A primary partner directs the preparation and distribution of the drug solution, usually keeping the cooker and cotton used to share drugs. The soaked filter and the drug residue left in the cooker can be later re-used adding a little bit of water for another shot. Usually, this role is occupied by the user that contributed the most to the caballo. These roles are not static, it is possible for one user to be a primary partner in one caballo but engage in another drug sharing arrangement later on, either with the same partner or a different one, without being in charge of the process.

In addition, partners might follow different strategies while seeking to jointly acquire and use drugs. “Fixers” do *caballo* with a limited number of trusted injection partners in their network, usually kin, or others with whom they have close relationships, from school age friends, to neighbors or those with whom they have shared drugs extensively in the past. By minimizing the number of partners and routinizing sharing expectations, this strategy ensures access to resources while limiting the potential problems associated with doing *caballo* with strangers. Other PWID take on the role of “maximizers,” entering into *caballo* with as many partners as possible, increasing their opportunities to access drugs by multiplying potential partners. Sometimes maximizers only know their caballo partners because they have seen them around, in Puntos, or shooting galleries, or because they have done a caballo in the past. The downside is that this choice also increases the potential problems associated with the transaction—robbery, cheating, hoarding.

Yet, neither of these are fixed strategies. A PWID might have been a maximizer but, over time, begun doing *caballo* with a limited number of partners, and the opposite also happens. Jail, drug treatment, quitting drug use, and migration can all affect a person’s social networks and their ability to engage in *caballo*. Of course, this is only an approximate typology, and some users are neither “fixers” nor “maximizers” but operate in between these extremes.

Whether users might be primary partner or not, or rely on a “fixer” or a “maximizer” strategy, they all seek in their interactions some kind of fair play, adhering to the norms that regulate drug sharing in the community. Bebe, in his late 30 s, washes cars at a local gas station in addition to taking turns as a drug dealer in the only drug selling spot in town. He explains the need for reciprocity:

“I tend to avoid caballo, if I have all the money I need for my dose I get the drug and I get your money and even if you don’t have enough, I share with you because I know what it is to be ‘sick.’ How much do you have, I would ask? Four dollars [an insufficient amount for a 50/50 caballo]. Fine! Let’s go! and we share. But then I remember that in other occasions it was me that had only four bucks and I were really sick and he had his full dose and he decided not to help me out, leaving me sick to fend for myself. I still decided to help him but I tell him right away: ‘see, before you told me you couldn’t help me because you had enough for yourself and now you are desperate, see how the world is round? Yesterday it was me [that needed] and today it’s you. Come here, I’ll fix it!’”.

*Caballo* partners who consistently demand more than their fair share are labeled “problematic” and tend to be excluded. “Tricksters” are also avoided. According to participants, tricks are very common among PWID, such as pocketing the money that participants have pooled to do *caballo*. This is viewed very negatively, not only because it causes a lack of trust among partners but also because it deprives users of the “cure” they need, forcing them to hustle for money again before they can have their dose. Tampering with “bags,” for example by taking a cut, is also negatively viewed but is judged less severely than the first situation. Participants also worry about others pulling a “water shoot,” a trick in which an injector uses deception to substitute drugs with water. A similar and more frequent trick involves placing not one but two cotton balls in the cooker, hiding the one used to filter the drug under the finger holding the cooker, and leaving the other with only a trace of drug for the other injector to use. Personality issues and past disagreements also play a role in selecting potential partners. Those users with more social connections will be able to find more suitable partners for a *caballo* while being able to reject those partners deemed less desirable. The reverse is also true: those users with fewer social connections might not have as many options. Caballo partnerships are often shaped by asymmetric dynamics involving gender and other forms of power disparities among prospective members. These in turn affect risk taking and drug related harms. Caballo partners try to manage HIV/HCV by serosorting, but most assume that there is no point in asking about their partners’ HIV status, because “they will lie to you.” Perhaps because they are in a more vulnerable position, women tend to ask their injection partners about their HCV status more often than men do. Women are also more likely to avoid entering in a caballo with somebody if they know the prospective partner has a positive status.

## Discussion

This study outlines how the social practice of *caballo* has contributed to the production of an HCV epidemic among PWID in rural Puerto Rico. Results show that 84.84% tested positive reactive to HCV, a result that has been confirmed by other epidemiological surveys in the area [43]. PWID tend to avoid direct sharing of syringes; only 7.14% reported having used a needle after somebody else had employed it, and 84.62% used a sterile needle the last time they used drugs with somebody. On the other hand, participants often engaged in indirect sharing: 71.43% divided drugs with a cooker or cotton that had been used by somebody else, and 32.14% divided drugs with a syringe that had been used by somebody else. These forms of indirect sharing are linked to the practice of caballo and the need to divide up jointly acquired drugs, which in turn increases the risk of HCV among this population. While PWID in our study tend to avoid direct sharing, indirect sharing is driven by the mode of drug acquisition, drug packaging and pricing, a reliance on speedball, the need to avoid heroin withdrawal, and a strict drug policy that encourage users to carry small amounts of illicit substances, among other factors.

The finding that participants attempt to manage HIV/HCV transmission risk during caballo by serosorting has been replicated in other studies of PWID [44–46]. However, the epidemic levels of HCV in this population suggest that this strategy has serious limitations and that individual behaviors alone are insufficient to curb HCV transmission. Findings illustrate the need to understand social epidemics such as HCV among PWID, leaving behind individual-centered models prevalent in public health. Decades of ethnographic studies with this population have shown that what appears to be the product of individual behaviors is better comprehended as a result of the particular social contexts in which PWID live and make decisions about risk [47–56]. Moving beyond public health’s emphasis on individual behaviors, the notions of “risk environment” [57] and “syndemics” [58] have recently been used to analyze the interplay of micro-level dynamics and larger structural forces in accounting for risk outcomes. While both approaches share some features, Rhodes (2009) suggests that a risk-environment approach—combining insights from political economy, social epidemiology, and the sociology of health—can be productive for understanding how the relationship between individuals and environment can produce drug harms, thus contributing to a social science of “harm reduction.” [59].

Our findings show that poverty and economic dispossession have been found to be an important driver of indirect sharing among PWID. High frequency users might spend $100 or even more a day, not an insignificant sum considering that Puerto Rico has a per capita income more than half of the poorest US States like Missouri or Mississippi [60]. The same relationship between poverty and drug sharing has been replicated in numerous studies [27, 31, 61]. In a study of homeless PWID in San Francisco Bourgeois (1997) finds that if users cannot afford their dose in full, indirect sharing allows users to regulate their dose intake in order to minimize the risk of experience heroin withdrawal, with some participants using half or even a third of a bag each time. In turn, using small doses encourages higher frequency of drug injection, making drug sharing more likely.

Our findings have shown that roles within drug sharing arrangements are not fixed. This aspect has been documented also by Finlinson [30] suggesting that roles change within the process of acquiring and dividing up drugs. According to this author, a primary partner in charge of dividing up the drug solution using their own equipment, might become a secondary partner receiving a drug solution that has been divided using up somebody else’s equipment in another caballo arrangement. These practices facilitate the transmission of HCV within a population, complicating harm reduction efforts.

One of the most distinctive features of indirect sharing in our study is that the drug preparation is usually loaded from the cooker using a single syringe, and then distributed to the injecting partners through backloading. While indirect sharing offers a fair method for equitable distribution [62], participants in our study do not seem to favor drawing the drug solution directly from the cooker, because of the perception that it makes it harder to ensure an equal share of the drug. It is possible that this preference might be driven by a lack of trust among injection partners and that in different social contexts, other ways of dividing up the drug solution might be preferable. A study of Puerto Rican PWID in New York and Puerto Rico show that PWID in New York City take turns to draw the drug solution from the cooker while those on the Island distributed it through backloading as well as taking turns [63]. The fact that our findings correspond not to metropolitan San Juan, but rural Puerto Rico might account for extensive use of backloading. As other authors have shown, the joint acquisition and use of drugs appear to be shaped by particular cultural norms, which in turn are an adaptive response to a particular risk environment [64].

Examining the social context in which PWID use drugs and make decisions about risk is critical for the successful adoption of harm reduction strategies to reduce HCV [65–68]. While harm reduction policies that emphasize the distribution of injection equipment are a valuable component in syringe exchange programs, findings suggest that preventing the sharing drug preparation equipment while sharing drugs might be more complex that behavioral health models suggests [69]. Indirect sharing occurs not because PWID lack information about HCV risk or do not possess knowledge about safe injection practices. This perspective has been criticized for blaming the victim [70] while obscuring the role of social and risk environments in shaping drug sharing arrangements [71, 72].

In addition, local implementation of effective harm reduction recommendations regarding HCV transmission among PWID is hampered by larger political and socio economic processes such as the Colonial status of Puerto Rico that deprives the Island of the resources it needs to tackle an HCV epidemic, while enacting an aggressive version of the war on drugs and subsequent incarceration that furthers the impact of HCV within its population [73]. Furthermore, the subjection to US patent laws and drug pricing ensures that despite the fact that a new HCV treatment is available (in the form of a three-month-long direct-acting antiviral regimen), the cost of that treatment is approximately $50,000. In the continental US, the cost of this treatment is covered by most private insurers or by Medicare/Medicaid; in Puerto Rico, few PWID have access to private insurance, and HCV treatment is not covered by La Reforma, a local version of Medicare/Medicaid for HIV-negative individuals who constitute the majority of the PWID population [74]. The extremely low HCV coverage among PWID [75] continues to fuel the epidemic as HCV-negative users are more likely to enter *caballo* arrangements with somebody infected by the virus. Expanded HCV treatment coverage would not only reduce the incidence of HCV among this population but also would contribute to provide certain measure of “herd immunity” protecting those that are HCV negative [76].

Similarly, access to harm-reduction-based interventions such as medically assisted treatment and syringe exchange provision have been shown to be protective against HCV transmission [77–83]. Unfortunately, these resources are severely lacking in Puerto Rico, particularly in rural areas [84]. A recent study showed that rural PWID have less access to syringe exchange in rural areas than those in urban settings, although both have had to resort to acquiring syringes from drug stores, peers, and drug dealers [85].

Understanding the social factors behind the HCV epidemic in Puerto Rico has a renewed urgency. As the Island was starting to recover from the aftermath of Hurricane Maria in September 2017 it was hit by the arrival of Covid-19, a pandemic with an epicenter in Wuhan China that has currently stricken most of the world. While the lasting effects of Covid-19 are not yet well known, it is likely the pandemic will reinforce the devastating effects of hurricane Maria which furthered a protracted economic crisis on the Island, while exposing deep political corruption and mismanagement practices, [86, 87] severely affecting the provision of health services PWID rely on, like Medically Assisted Treatment and Syringe Exchange Providers, leading the way to the worsening of an already existing HCV epidemic.

Increasingly, harm reduction programs will need to consider the impact of large-scale natural disasters, or pandemic events, and its effects on the risk environment of PWID, already afflicted by severe forms of poverty, social suffering, and structural violence [88].

One limitation of this study is that findings are based on a sample that is overwhelmingly male. While this distribution mirrors not only the composition of the parent study but also of other studies of PWID in Puerto Rico that seem to be gendered, the relative lack of female participants raises questions about how gender dynamics and power imbalances might shape drug sharing arrangements among PWID in rural Puerto Rico. More research is needed to explore this issue. We believe that despite this limitation, the study of the social organization of drug sharing arrangements makes an important contribution to the understanding of HCV risk among this population.

## Conclusions

This study shows that drug sharing plays an important role in the HCV transmission among PWID in rural Puerto Rico. While participants avoided direct sharing of syringes, they were forced to share the cooker and cotton used to divide and inject the drug solution. Drug sharing occurs not only within the joint acquisition and use of injection drugs, but in a particular risk environment that contributes to HCV risk. Poverty, drug packaging and pricing, a reliance on speedball, the need to avoid heroin withdrawal, and a strict drug policy that encourage users to carry small amounts of illicit substances, among other factors fuels the HCV transmission. This finding complicates harm reduction interventions based on the distribution of injection equipment or information about safe injection practices alone, suggesting that preventing indirect sharing practices should consider the social context in which PWID acquire and use drugs.

## Data Availability

The datasets used/or analyzed during the current study are available from the corresponding author on reasonable request.

## Abbreviations

HCV: Hepatitis C
HIV: Human immunodeficiency virus
IRB: Institutional Review Board
NHBS: National HIV Behavioral Surveillance
US: United States
